# 

**Published:** 1850-01

**Authors:** 


### INDEX.

A

Absorption of the testes from metastasis of mumps 588
Abstract, Half-yearly, of the Medical Sciences. By Dr. Ranking, bib. notice of 238
Acclimation, inaugural essay on 439, 499
Acetic acid, as a prophylactic in scarlatina 560
Acid, sulphuric, fraudulent uses of 70
Acid, tannic 498
Aconite in dysentery 187
African cranium, observations on, by Dr. Jno. Neill, 57
Air, prevention of entrance of, into cavities of abscesses, &c. 135, 254
Alcohol, changes of, when taken into the circulation, 496
Aloetico-febrifugic elixir of Recamier 185
American Medical Association 174, 357, 423, 709
American Medical Association, delegates to 118
American Medical Association, transactions of 230
Anatomy and physiology 53, 121, 176, 429, 548, 618, 666
Anatomy and physiology, contributions to 202
Anatomy, surgical. By J. Maclise, Surgeon, bib. notice of 584
Ancell on the peculiarities of structure in persons predisposed to tubercles 553
Aneurism, popliteal 682
" needle 682
Antispasmodic, a new 437
Applications, pharmaceutical and toxicological, of carbon 64
Appointments and changes 51
" medical 424
" of Dr. E. N. Horsford 664
" of Drs. S. D. Gross, and Elisha Bartlett, 613
Appointment of Dr. T. O. Edwards, 487
Army Medical Board 248
" order, 616
Arrested muscular developement, case of 453
Arsenic, case of poisoning by 619
" influence of, on digestion 619
Artery femoral, unsuccessful ligation of 67
Artery, femoral, ligation of in popliteal aneurism, 682
Asiatic cholera, report of the committee of internal health on, Boston, 1849, bib. notice of, 399
Assimilated rank in navy, 358, 424, 484
" " history of the existing controversy on the subject of; bib. notice of, 657
Astley Cooper Prize 425
Asylums, Insane 119
Auscultation, intestinal, Essay on. By Chas. Hooker, M. D., bib. notice of 37
Autoplasty in gangrene of the scrotum 66
Auscultatory signs of enlarged liver, 731

B

Baltimore Medical and Surgical Journal 485
Bartlett, E. D., appointment of 613
Batchelder on inflammation, 102
Baths, a treatise on, including cold, sea, warm, hot, vapor, gas, and mud baths ; on the watery regimen, hydropathy, and pulmonary inhalation ; with a description of bathing in ancient and modern times. By J. Bell, M. D., bib. notice of 289
Baxley, Dr., appointment of 613
Bellingham on the impulse of the healthy heart, 429
Benson on Cod-liver oil 559
Bernard's discovery of the renal circulation, exposition of 549
Bibliographical notices 26, 102, 154, 220, 277, 343, 397, 455 542, 584, 646, 709
Bile of the ox, decomposition of 181
Bitterness of quinia, removal of 498
Blood vessels arrangement of in the mucous membrane of the stomach, 733

Blood, white, case of 495
Board, army medical, 248
Bodies foreign, removal of, from ear passages, 318
Body, structure, in tuberculous predisposition, 553
Bones, a treatise on diseases of. By E. D. Stanley, F. R. S., &c., bib. notice of 30
Bones of the African cranium, observations on. By Dr. J. Neill 37
Boy, lithotomy in a young 556
Bofareira, on the use of, as a means adopted by the natives of the Cape de Verde Islands to excite lactation, 728
Brachial artery, obliteration of 684
British and Foreign Medico-chirurgical Review, July, 1850, bib. notice of 612
Broma and chocolate 65
Bronchocele of newborn infants, 732

C

Cæsarian operation 524
Carbon, the pharmaceutical and toxicological applications of 64
Carious ribs, successful operation for the Exsection of 75
Case of placenta prævia 135
Catalogue of skulls of man, and the inferior animals, in the collection of Samuel George Morton, M. D., Penn., and Edinb., President of the Academy of Natural Sciences of Philada., &c., bib. notice of 114
Causes of inflammation, philosophically considered, 102
Case of idiopathic tetanus, by D.G. Gregory, M. D., 702
Changes and appointments, 57
Changes which ether and alcohol undergo when taken into the circulation 496
Charge to the graduates of Jefferson Medical College of Philadelphia, delivered at the public commencement, held March 9th, 1850. By J. K. Mitchell, M. D., bib. notice of 240
Cheiloplastic operation 13
Chemico-gelatinous injection, account of, with the method of employing it in anatomical preparations. By H. Goadby, Esq. 176
Chemistry, Encyclopædia of, Practical and Theoretical: embracing its application to the Arts, Metallurgy, Mineralogy, Geology and Pharmacy. By James C. Booth, A. M., assisted by Campbell Morfitt, bib. notice of 293
Chemistry, Medical, a practical handbook of. By Jno. E. Bowman, Fellow of the Chemical Society, author of "Practical Chemistry," &c., bib. notice of 611
Chilblains, treatment of, by M. Ossieur 64
Child, diminution of the size of, by regimen and venesection, 195
Chloroform, employment of, by thieves 316
Chocolate and broma 65
Choice extract 242
Cholera 174
" influence of, upon pregnancy and the fœtus 256
Chronic farcy, 96
Clavicles, fracture of both 19
Clinical remarks and cases, by Dr. Geo. McClellan, 687
Coagula, endo-cardial 389
Cœcum, ulceration of 100
Cod-liver oil, probable danger from the use of 559
Coffee, nutritive properties of 558
Colchicum in dropsy 128
Collodion in gangrene of the scrotum 66
Commencement of the winter sessions 613
Confectionary, colored, case of poisoning by 434
Contributions to Physiology. By Bennett Dowler, M. D., of New Orleans, bib. notice of 42
Contributions to anatomy and physiology, by W. R. Handy, M. D. 202
Contributions to obstetrics, with tabular views and miscellaneous practical observations. By H. A. Ramsay, M. D. 562
Contributions for the revision of the pharmacopæia 118
Copland on the causes, nature, and treatment of palsy and apoplexy; of the forms, seats, complications, and morbid relations of paralytic and apoplectic diseases, bib. notice of, 723
Correspondence between Dr. C. J. B. Williams, and a Homœopathic practitioner 426
Correspondents 248
Cough, nervous or convulsive 127

Croup, treatment of, by calomel and alum, 727
Crystalline lens, dislocation of from a blow 141
Cyanosis, Walter's case of 340

D

Deaths in Philadelphia, 249, 317, 427, 665, 488, 547, 617, 665,
Death of Marcus L. Taft, M. D., 256
" Amos Twitchell, M. D., 424
" R. E. Griffith, M. D., 428
" President of U. S. 486
" J. T. Shotwell, M. D., 487
" Dr. Hartshorne 614
" Sam'l B. Woodward, M. D. 119
" Jno. F. Brooke, M. D., U. S. N. 119
" S. M. Kane, M. D., 119
Decomposition of the bile of the ox 181
Delegates to the American Medical Association 118
Delegates to the State Society 118
Delegates to the convention for revising the pharmacopœia, 118, 173, 246
Demonstrative midwifery 614
Dental Surgery, principles and practice of. By C. A. Harris, M. D. &c., bib. notice of 542
Development, arrested muscular, case of 453
Development of muscular fibres of the heart, 433
Diagnosis of pregnancy, 255
" difficulties of, in certain diseases, 679
Diseases of the eye, lectures on, 726
Diagnosis, Pathology and Treatment of the Diseases of the Chest. By W. W. Gerhard, M. D. &c., bib. notice of 595
Dietetical and Medical Hydrology, a Treatise on Baths, &c. by John Bell, M. D., &c., bib. notice of 289
Digestion, influence of arsenic upon 619
Diminution of the size of the child by regimen and venesection 195
Discussion on the power of quinia in remittent fevers 78
Diseases of the Bones, a Treatise on, by E. Stanley, F. R. S., &c., bib. notice of 30
Diseases of Infants and Children. By Chas. D. Meigs, M. D., &c., bib. notice of 646
Diseases of Females, including those of pregnancy and childbed. By Fleetwood Churchhill, M. D., &c., bib. notice of 287
Dislocation of the crystalline lens from a blow 141
Dislocation of the Shoulder, a Treatise on. By G. O. Jarvis, M. D. &c., bib. notice of 158
Distortion of the face and neck from a burn 188
Dogwood, swamp, case of poisoning by 641
Dowler's contributions to physiology 42
Dowler's researches into the natural history of death 598
Dropsy, colchicum in 128
Druggists' general Receipt book: containing numerous receipts for patent and proprietary medicines, druggists' nostrums, etc. By H. Beasely, bib. notice of 295
Duclos, on the use of nux vomica in impotence and spermatorrhœa, 62
Dysentery, aconite in 187
" injections of arg. nitras in 252

E

Ear passages, removal of foreign bodies from 318
Editorial 51, 118, 171, 242, 298, 357, 423, 483, 545, 613, 662
Education medical, report on 230
Edwards, Dr. T. O., appointment of 487
Elongation of under jaw, caused by a burn 188
Elementary chemistry, theoretical and practical, by Geo. Fownes, M. D., edited with additions by Robert Bridges, M. D., bib. notice of, 725
Emmenagogue properties of polygala senega 627
Emerson on the comparative mortality of children 147
Emphysema of the sub-mucous cellular coat of the stomach 205
Employment of chloroform by thieves 316
Encyclopædia of Chemistry, Practical and Theoretical, &c. By J. C. Booth, A. M., &c., bib notice of 293
Endo-cardial coagula 389
Epidemic dysentery, nitrate of liver in 450
Epilepsy, valerian in 63
Ergotine in external and internal hemorrhages 625
Essays on the opium trade, including a sketch of its history, &c. By N. Allen, M. D., bib. notice of 346
Essays on the Puerperal Fever and other Diseases peculiar to Women. By F. Churchill, M. D., &c., bib. notice of 469
Essay on Intestinal Auscultation. By Charles Hooker, M. D., &c., bib. notice of 37
Esophagus, self-inflicted wound of 489
Ether, changes of, when taken into the circulation 496
Etiology, Pathology and Treatment of Congenital Dislocations of the head of the femur, a Treatise on. By J. M. Carnocham, M. D., &c. bib. notice of 277
Excision of the os maxillare superius 16
Excision of the os calcis 438
Exeter, Cholera in, History of. By Thos. Shapter, M. D., &c., bib. notice of 226
Experimental inquiry into the causes of the sounds of the heart, 53
Experimental researches on the action of quinia 131
Exposition of Bernard's discovery of the renal circulation 549
Exsection of two carious ribs and lower part of the sternum 75
External manipulation, case of turning by 625
Extirpation of the Testicle 254
Extra-uterine pregnancy, 511, 638

F

Face, distortion of, from a burn 188
Facts vs. figures 298
Farcy, chronic, case of 96
Females, diseases of: including those of Pregnancy and Childbed. By F. Churchill, M. D., &c., bib. notice of 287
Femoral artery, ligation of, for popliteal aneurism 682
Femoral artery, ligation of, for wounds of the anterior tibial 67
Femur, fractured, death from 683
Flint on injections of arg. nitras and creosote in dysentery 252
Fiske Fund Prize Dissertation. By H. G. Clarke, M. D., &c., bib. notice of 397
Fits, hysterical, moral treatment of 255
Fœtus, influence of cholera upon 256
Foramen thyroid, Dr. Neill on 618
Foreign bodies in the ear passages 318
Formulary, a Universal, containing the methods of preparing and administering officinal and other medicines, &c. By R. E. Griffith, M. D., &c., bib. notice of 220
Fownes' Elementary Chemistry, bib. notice of, 725
Fracture of both clavicles 19
Fracture compound, of the humerus 684
France, Military Surgeons of 615
Fraudulent uses of sulphuric acid 70
Friendly hints to a reviewer 526
Frick on renal affection, their diagnosis and pathology, bib. notice of, 724

G

Gangrene of the scrotum 66
" pulmonary 333
Gastric juice, changes effected by on nitrogenized and non-nitrogenized food 182
Gastrotomy, case of 630
General Therapeutics and Materia Medica: adopted for a Medical Text Book. By R. Dunglison, M. D., &c., bib. notice of 543
Gentle hint to correspondents 248
Graduates and students 245, 310
" Proportion of, to the population 199
Graduates of Jefferson Medical College, charge to 246
Grafton on Ship-fever 397
Gregory, Dr., case of idiopathic tetanus, by 707
Griffith's universal formulary 220
" death of 428
Gross, Dr. S. D., appointment of 63

H

Half-yearly Abstract of the Medical Sciences, bib. notice of 238
Hampden Sidney College 310
Hartshorne, Dr., death of 614
Hay fever, cause and cure of 677
Homœopathy condemned by law 485
Homœopathic correspondence 426
Horsford, Dr., E. N., appointment of 664
Horner, Dr., remarks on the medical topography of Callao and Lima, 694
Hospital cases 644
" St. Joseph's 130
Household surgery, or hints on emergencies. By John F. South, one of the surgeons of St. Thomas's Hospital, bib, notice of, 724
Hæmatemesis, treatment of 621

Head of the femur, congenital dislocations of 277
Heart, impulse of 429
" inquiry into the sounds of 53
Hemorrhage from ulcerated cœcum 100
" use of ergotine in 622
Heart, structure of the muscular substance of, 734
Hints to a reviewer 526
History, nature and best treatment of Ship-fever by H. Grafton, M. D &c., bib notice of 397
History, Natural, of Death. researches into. By B. Dowler, M. D., &c., bib. notice of 598
History of the controversy on the subject of Assimilated Rank in the Navy of the U. S. By W. S. W. R., bib. notice of 657
History of the Cholera in Exeter in 1832. By T. Shapter, M. D., &c., bib. notice of 226
Human Physiology, Principles of, with their chief application to Pathology, Hygiene, and Forensic Medicine. By Wm. B. Carpenter, M. D., &c., bib. notice of 35
Human Physiology. By Robley Dunglison, M. D., bib. notice of 610
Hydrocele of the neck 257
Hydrology, Dietetical and Medical. By Jno. Bell, M. D., &c. bib. notice of 289
Hydrophobia, remedy for 132
Hydrostatic, principles in explanation of the sensibility of the teeth 584
Hysterical fits, moral treatment of 255

I

Illustrations of the difficulties of diagnosis in certain forms of diseases 679
Impotence, use of nux vomica in 62
Impulse of the heart, 429
Inaugural essay on acclimation 439
Incontinence of urine in children 678
Infants and children, diseases of. By F. Churchhill, M. D., &c., bib. notice of 26
Infection, purulent, case of 275
Inflammation: its Symptoms, Causes and Treatment, Philosophically considered. By J. P. Batchelder, M. D., &c., bib. notice of 102
Inflammation of the Uterus and its appendages, a Practical Treatise on. By J. H. Bennett, M. D., &c., bib. notice of 343
Influence of dry and moist air in the causation of disease 319
Infants, newborn, bronchocele of 732
Ingrowing of the toe-nail 623
Injection, chemico-gelatinous 176
Injury resulting in peritonitis 674
Insane asylums 119
" Report of the Pennsylvania Hospital for the, for the year '49. By T. S. Kirkbride, M. D., bib. notice of 239
Interior Valley of North America: a systematic Treatise on the diseases of. By D. Drake, M. D., &c., bib. notice of 348
Intermittent fever, use of strychnia in 382, 523
Intestinal Auscultation, Essay on. By Chas. Hooker, M. D., bib. notice of 37
Intestines, wound of 146
Introductory Lectures 245
Ipecacuanha, practical remarks upon 623

J

Jackson's reply 526
Jaw, elongation of, 188
Jefferson Medical College, charge to graduates of. By J. K. Mitchell, M. D., &c., bib. notice of 240
Johnson on extra-uterine pregnancy 511
Johnson's pathological cases 379
Journal, Southern Medical and Surgical 664
Journal, St. Louis, Medical and Surgical 310
Jurisprudence, Medical. By A. Taylor, F. R. S., &c., bib. notice of 544

K

Kane, Dr. S. M., death of 119
Knife, Lane's, in division of the rectus in strabismus 492
Kousso in expulsion of tape worm 494
Knox on the races of men, bib. notice of 716

L

Lacerated wounds, use of arg. nitras in 133
Lactation in the male, case of 454
Lane's knife in division of the rectus, in strabismus 492
Lectures on the Processes of Repair and re-production after injuries. By James Paget, F. R. C. S., bib. notice of 121
Lepra vulgaris, treatment of 18

Library, students' and medical practitioners' 246, 295
Liebig, visit of 484
Ligation of the femoral artery for a wound of the anterior tibial 67
Lithotomy in a very young boy 556
Liver enlarged, auscultatory signs of, 731
London, Sydenham Society of 545
Lectures on diseases of the eye, 726

M

Maclise's Surgical Anatomy, bib. notice of 487
Male, lactation in, case of 454
Manipulation, external, in turning 625
Marbot on aconite in dysentery 187
Marcus L. Taft, M. D., death of 246
Materia medica and therapeutics 62, 131, 185, 437, 498, 558
Materia medica and general therapeutics. By R. Dunglison, M. D., &c., bib. notice of 543
Materia Medica and therapeutics. By T. D. Mitchell, M. D., &c., bib. notice of 589
M'Clellan, Dr., cases and clinical remarks by 607
Medical Association, American, delegates to 118
Medical Society of the State of Pennsylvania 120, 247
Medical Formulary. By Benj. Ellis, M. D., &c., bib. notice of, 220
Medical practitioners' and student's library 246
Medical schools, summer 247
" graduates and students 310
" appointments 424
" department of army 428
" and Surgical Journal Baltimore 485
Medical faculty of the university of New York 485
Medical jurisprudence. By A. Taylor, F. R. S., &c., bib. notice of 554
Medico-chiurgical Review, British and Foreign, July, 1850, bib notice of 612
Medical topography of Callao and Lima, remarks on, by Dr. G. R. B. Horner, 694
McDougal on ingrowing of the toe-nail 623
Medicinal extracts 173
Melœna, treatment of 621
Membrana tympani, structure of 673
Meningitis, tubercular, case of 130
Menstruation, vicarious 74
Men, the races of, by Robert Knox, M. D., bib. notice of 716
McWilliams, on the use of the bofareira, as a means of exciting loctation, 728
Midwifery, a Theoretical and Practical Treatise on, including diseases of pregnancy and parturition. P. Cazeaux, adjunct Prof., &c. Translated by R. P. Thomas, M. D., &c., bib. notice of 154
Military surgeons in France 615
Mill's case of death from fractured femur 583
Moral treatment of hysterical fits 255
Mortality of male children, causes of 147
Mortuary statistics 317, 427, 488, 547, 617, 665
Mott, Dr., resignation of 485
Mucous membrane of intestinal canal in cholera, report on condition of 143
Muscular fibres of the heart, researches on the development of 433

N

National Medical Convention for revising the Pharmacopœia of the U. S. 376
Natural history of death, researches into. By B. Dowler, M. D.' &c., bib. notice of 598
Nature of gastric juice, and changes, its effects on nitrogenized and non-nitrongenized articles of food 182
Native properties of coffee 552
Navy, assimilated rank in 302, 358, 484
Neck as a medical region in syncopal seizure, &c. 57
Neck, hydrocele of 257
Neligan on the treatment of Hæmatemesis and Melœna 621
Neill on the sensibility of the teeth 548
Nervous and convulsive cough 127
Neuralgia, treated by cold draughts after sweating 253
New adhesive dressing 559
New aneurism needle 684
New antispasmodic 437
New method of operating for extirpation of the testicle 234
New York, meaical faculty of university of 485
Nitrate of silver in dysentery 252, 450
" " lacerated wounds 133
Nux vomica in hay fever 677

Nux vomica in impotence and spermatorrhœa 62

O

Obesity simulating pregnancy 225
Obliterated brachial artery 684
Observations in certain diseases of young children. By Chas. D. Meigs, M. D., &c., bib. notice of 646
Observations on emmengogues 627
" " endo-cardial coagula 389
Obstetrics, contributions to 562
Occipital and superior maxillary bones of the cranium, observations on. By Dr. John Neill 57
Oleum terebinthinæ in typhus fever 208
Operation, cæsarian 524
" cheiloplastic, case of 13
" for extirpation of the testicle 254
Opium trade, essay on. By N. Allen, M. D., &c., bib. notice of 346
Original communications 13, 75, 143, 199, 257, 319, 379, 439
Os calcis, excision of 438
Os maxillare superius, excision of, by a modified operation, 16
Osseous deposition in nervous pulp of a molar tooth 637
Ossieur on treatment of chilblains 64
Ovarian dropsy 630
" tumor, removal of 485
Ovariotomy 248
Ox, decomposition of bile of 181

P

Pathological cases 379
Pathology and practice of medicine 57, 127, 252, 434, 494, 555
Palsy and apoplexy, the causes, nature, and treatment of, and of the forms, seats, complications, and morbid relations of paralytic and apoplectic diseases. By James Copland, M. D., &c., bib. notice of, 723
Peculiarities of structure diagnostic of tuberculous predisposition 553
Pennsylvania hospital: surgical wards 21, 625, 705, 706, 707, 708
Pennsylvania State Medical Society 310
" University of 378
People vs. Dr. H. N. Loomis, for libel 544
Pritonitis resulting from injury 674
Placenta prævia, case of 135
Plugging the vagina with vulcanized caoutchouc 194
Pharmaceutic applications of carbon 64
Pharmacopœia, National Convention for revision of 118
Pharmacopœia, delegates to Convention for revision of 118, 173
Philadelphia County Medical Society, discussions of 78, 147, 208, 319
Physiology and anatomy 53, 121, 176, 429, 548
Physiology, contributions to. By B. Dowler, M. D., bib. notice of 42
Poisoning with arsenic, case of 619
" " berries of the swamp dogwood 638
Physiology, Human. By R. Dungli-son, M. D., &c., bib. notice of 610
Poisoning with colored confectionary 434
Polygala senega, observations on the emmenagogue properties of 627
Popliteal aneurism 682
Practical Handbook of Medical Chemistry. By Jno. E. Bowman, F. C. S., &c., bib. notice of 611
Practical remarks on Ipecacuanha 623
Pregnancy, caution in diagnosis of 255
" influence of cholera on
" case of 492
" extra-uterine 511, 638
Prevention of the entrance of air, in removing fluid from absesses, &c. 135, 254
Prevention of the scarlatina by acetic acid 560
Principles of human Physiology, with their chief applications to pathology, hygiene and forensic medicine. By Wm. B. Carpenter, M. D., &c., bib. notice of 35
Principles and practice of dental surgery. By C. A. Harris, M. D., &c., bib. notice of 542
Prize, Astley Cooper 425
Proceedings of the American Medical Association, at the third annual meeting 359
Professorial changes and appointments 309
Prolapsus uteri during parturition 194
Properties of coffee 558
Proportion of graduates to the population 199
Psoriasis, treatment of 186
Puerperal fever and other diseases peculiar to women, Essays on.

By F. Churchhill, M. D., &c., bib. notice of 469
Pulmonary gangrene 333
Purulent infection, 275

Q

Quinia, experimental researches on the action of 131
Quinia in remittent fevers 78
" removal of bitterness of 498

R

Rank of army and navy officers 616
Recamier's aloetico-febrifugic elixir 185
Record of Medical Science, 53, 121, 171, 252, 429, 489
Rectus, division of, in strabismus 492
Regimen and venesection in diminishing the size of the child 195
Remedy for hydrophobia 132
Remittent fevers, quinia in 78
Removal of an ovarian tumor 485
Removal of foreign bodies from the ear passages 318
Renal circulation, exposition of 549
Renal affections, their diagnosis and pathology, by Charles Frick, M.D., bib. notice of 724
Repair and reproduction after injuries, lectures on the processes of 121
Reply, Jackson's 526
Report of a trial 544
Report of the Committee on internal health, on the Asiatic Cholera, &c. Boston, 1848, bib. notice of 399
Reports, Southern Medical. Edited by E. D. Fenner, M. D., &c., bib. notice of 406
Report of the Committe appointed to examine into the condition of the gastro-enteric mucous membranes of persons dying of cholera 143
Researches on the action of quinia 131
Researches on the development of the muscular fibres of the heart 433
Researches on the Natural History of Death. By B. Dowler, M. D., &c., bib. notice of 598
Resignation of Prof. Dudley 484
" of Prof. Mott 485
Retrospect of the progress of microscopic investigations, &c. 566
Reviewer, friendly hints to 526
Rheumatism, acute, treatment of 574
" treated by cold draughts after sweating 253
Rupture of the spleen 20

S

Sandras on nervous and convulsive cough 127
Scarlatina, prevention of, by acetic acid 560
Scarlatina, treatment of 98
Scrotum, gangrene of 66
Sensibility of the teeth explained on hydrostatic principles 584
Ship Fever, so called: its history, nature, and best treatment. By H. G. Clark, M. D., &c. bib. notice of 397
Silver, nitrate of, in lacerated wounds 133
Singultus, treatment of, by sulphuric acid 621
Skulls of man and the inferior animals, catalogue of. By Sam'l G. Morton, M. D., &c. bib. notice of 114
Spermatorrhœa, nux vonica in 62
Spleen, rupture of 20
Society of State of Pennsylvania, Medical 120
Sounds of heart, inquiry into causes of 53
Southern Medical Reports. Edited by E. D. Fenner, M. D., &c. bib. notice of 406, 455
Southern medical students 662
" Medical and Surgical Journal 664
South's Household Surgery, or hints on emergencies, bib. notice of 724
Steiner's hospital cases 634
St. Louis Medical and Surgical Journal 310
Stomach, emphysema of submucous cellular coat of 205
Strabismus 492
Stricture of the urethra, memoir on. By J. P. Mettauer, M. D., &c., bib. notice of 404
Structure of the membrana tympani 673
Students and graduates 245, 523
Strychnia in intermittent fever 382, 523
Structure of the muscular substance of the heart, 734
Sulphuric acid, fraudulent uses of 70
Sumbul 437
Summer medical schools 247
Superior maxillary bones of the cranium, observations on 57
Surgery 66, 133, 188, 254, 438, 489
Surgical anatomy. By Jno. Maclise, surgeon, bib. notice of 40, 292, 584
Surgical cases 38

Surgical uses of vulcanized india-rubber 559
Swamp dogwood, case of poisoning by 641
Sydenham Society 171, 545, 727
Symptons, causes and treatment of inflammation, philosophically considered. By P. Batchelder, M. D., &c., bib. notice of 102
Syncopal seizure 57
Systematic treatise, historical, etiological, and practical, of the principal diseases of the interior valley of N. America, &c. By D. Drake, M. D., &c., bib. notice of 348

T

Tape-worm, expulsion of by kousso 494
Testes, absorption of 588
Testicles, extirpation of 254
Theoretical and practical treatise on midwifery, including the diseases of pregnancy and parturition. By P. Cazeaux, adjunct professor, &c., translated by R. P. Thomas, M.D. bib. notice of 154
Therapeutics 62, 185, 498
" and Materia Medica, general, adapted for a medical text book. By R. Dunglison, M. D. &c. bib. notice of 543
Three Lectures, preliminary to a course on the principles and practice of surgery. By Wm. Gibson, M. D., &c., bib. notice of 169
Thyroid foramen in the male and female innominatum 618
Tobacco, influence of 614
Toe-nail, ingrowing of 623
Toxicological applications of carbon 64
Transactions of the American Medical Association 161, 709
Treatise on diseases of the bones. By E. Stanley, F. R. S., &c., bib. notice of 30
Treatise on the diseases and physical education of children. By Jno. Eberle, M. D. Edited by Thos. D. Mitchell, M. D. &c., bib. notice of 646
Treatise on dislocation of the shoulder. By Geo. O. Jarvis, M. D., &c. bib. notice of 158
Treatment of acute rheumatism 574
" of chilblains 64
Treatment of hematemesis and melæna 621
Treatment of psoriasis and lepra vulgaris 186
Treatment of scarlatina 98
" of croup by calomel and alum, 727
Tubercular meningitis 130
Turning by external manipulation 625
Typhus fever, ol. terebinthinæ in 208

U

Ulceration of the cœcum 100
Under-jaw, elongation of 188
Universal formulary, &c. By R. E. Griffith, M. D., &c., bib. notice of 220
University of New York 247
" of Pennsylvania 378
Urethra, a memoir on stricture of. By J. P. Mettauer, M. D., &c., bib. notice of 404
Urine, incontinence of, in children 678
Uterus and its appendages, practical treatise on, &c. By J. H. Bennett, M. D., &c., bib. notice of 343
Uterus, prolapsion of, during parturition 194
Utility of acetic acid in prevention of scarlatina 559

V

Vagitus uterinus 197
Valerian in epilepsy 63
Vicarious menstruation 74
Vidal on placenta prævia 135
Visit of Liebig 484
Vulcanized india-rubber, surgical uses of 559
Vulgaris, lepra, treatment of 186

W

Waters on cyanosis 340
Western Journal of Medicine and Surgery, 725
"White-blood," case of 495
Williams on acclimation 499
Wills' hospital 642
Woodward, Dr. S. B., death of 119
Wound of intestines 146
Wounds, lacerated, nitrate of silver in 133
Wright on changes of alcohol and ether when taken into the circulation 496

### NOTICE TO CORRESPONDENTS.

Communications and Books for notice should be addressed to the Editor, care of Messrs. Lindsay & Blakiston.

Letters, &c., connected with the *business affairs* of the Journal should be addressed to the Publishers.

Papers for publication must be received *before* the 20th of the month, or they cannot appear in the forthcoming number.

The following Journals and Publications have been received since Dec. 1st:

The Boston Medical and Surgical Journal. (Weekly, Boston.)
Buffalo Medical Journal.
Western Journal of Medicine and Surgery.
Medical News.
Western Lancet.
Transylvania Medical Journal.
Southern Medical and Surgical Journal.
American Journal of Sciences and Arts.
The British American Journal of Medical and Physical Science. (Montreal.)
The London Lancet. (Weekly, London.)
The Medical Times. (Weekly, London.)

---

Communications have been received from:—

R. G. Meredith, M. D., Canterbury, Va.
J. T. Hearne, M. D., Lowndsboro, Ala.
J. H. Weir, M. D., Philadelphia.
H. A. Ramsay, M. D., Raysville, Ga.
T. E. Waller, M. D., Camden, Del.
G. W. Brown, M. D., Port Carbon, Pa.
E. C. Banks, M. D., Lawrenceville, Ill.
D. B. Hilliard, M. D., Halifax Co., N. C.

---

The foreign correspondents of the Examiner will please direct their Exchanges and other communications to the care of Mr. Charles J. Skeet, 27 King William St., Charing Cross, London, or Mr. H. Bossange, 21 Bis, Quai Voltaire, Paris.

